# Kainic Acid-Induced Seizures Modulate Akt (SER473) Phosphorylation in the Hippocampus of Dopamine D2 Receptor Knockout Mice

**DOI:** 10.1007/s12031-012-9927-x

**Published:** 2012-11-29

**Authors:** Mark Dunleavy, Giovanni Provenzano, David C. Henshall, Yuri Bozzi

**Affiliations:** 1 Department of Physiology and Medical Physics, Royal College of Surgeons in Ireland, Dublin, Ireland; 2 Laboratory of Molecular Neuropathology, Centre for Integrative Biology (CIBIO), University of Trento, Via delle Regole 101, Mattarello, 38123 Trento, Italy; 3 CNR Neuroscience Institute, Pisa, Italy

**Keywords:** Epilepsy, Dopamine, Apoptosis, Wnt signalling

## Abstract

Dopamine D2 receptor (D2R) signalling has been shown to modulate seizure-induced hippocampal cell death. D2R knockout (D2R−/−) mice are more susceptible to kainic acid (KA)-induced excitotoxicity, displaying cell death in the CA3 subfield of the hippocampus at KA doses not damaging in wild-type (WT) animals. Absence of D2R signalling in the hippocampus leads to activation (dephosphorylation) of glycogen synthase kinase 3β (GSK-3β) after KA (20 mg/kg), which is not associated with a change in the phosphorylation of the GSK-3β regulator Akt at the canonical threonine 308 residue. In the present study, we investigated alternative pathways responsible for the activation of GSK-3β in the hippocampus of the D2R−/− mice 24 h following KA-induced seizures. Here, we show that phosphorylation of Akt occurs at serine 473 (Ser473) in the CA3 region of WT but not D2R−/− mice following KA. Moreover, the CA1 subregion, which does not undergo neurodegeneration in either WT or D2R−/− mice, displays a strong induction of Akt (Ser473) phosphorylation after KA. Additionally, the vulnerability in the CA3 is not associated with changes to p38MAPK and Dishevelled activation, and β-catenin does not appear to be a downstream target of the GSK-3β. Thus, we propose that GSK-3β phosphorylation-mediated hippocampal cell survival may depend on Akt (Ser473) phosphorylation; loss of D2R-mediated signalling in the CA3 region of D2R−/− mice leads to reduced Akt (Ser473) phosphorylation rendering neurons more vulnerable to apoptosis. Further investigation is required to fully elucidate the GSK-3β targets involved in D2R-dependent response to excitotoxicity.

## Introduction

Traditionally thought of as an imbalance of excitatory glutamate and inhibitory GABAergic transmission resulting in excitotoxicity, seizure-induced cell death has also been shown to be modulated by dopaminergic signalling (Bozzi and Borrelli 2006; Starr 1996). Mice lacking the dopamine D2 receptor (D2R−/− mice) (Baik et al. 1995) are more susceptible to kainic acid (KA)-induced excitotoxicity, displaying neurodegeneration in the CA3 subregion of the hippocampus at KA doses not epileptogenic for wild-type (WT) mice (Bozzi et al. 2000).

The pathway through which D2Rs modulate seizure-induced neuronal death in the hippocampus remains to be elucidated. The canonical pathway of D2R activation in neurons involves G-protein coupled downregulation of adenylate cyclase and subsequent cAMP downregulation (Bozzi et al. 2011). More recent studies in the striatum, however, suggest a role for cAMP-independent signalling involving a complex formed by D2R, protein phosphatase 2A, β-arrestin 2 and Akt (Bozzi et al. 2011). When activated by dopamine, the D2R causes the inactivation (dephosphorylation) of Akt at the threonine 308 residue (Thr308) and subsequent activation (dephosphorylation) of glycogen synthase kinase 3β (GSK-3β) (Beaulieu et al. 2011). These studies focus mainly on signalling in the striatum, with few studies investigating the cascades downstream of the D2R in the hippocampus. Terminal deoxynucleotidyl transferase dUTP nick end labelling staining, along with Bax and caspase 3 upregulation, has been reported in the CA3 hippocampal subfield of D2R−/− mice following KA treatment (Bozzi et al. 2000; Tripathi et al. 2010) indicating neuronal apoptosis. This was also associated with a downregulation (activation) of GSK-3β phosphorylation at serine 9 (Ser9) residue. Interestingly, these changes in GSK-3β were not associated with a change in activation of pAkt (Thr308), suggesting that an alternative pathway is responsible for the activation of GSK-3β in the hippocampus of the D2R−/− mice.

Recent research proposes p38 mitogen-activated protein kinase (p38MAPK) as a potential regulator of GSK-3β activity, by direct phosphorylation at its C-terminus (Thornton et al. 2008). Furthermore, GSK-3β is known to be an integral step in the Wnt signalling pathway. Following activation of Wnt receptors, Dishevelled (Dvl) is activated thus leading to GSK-3β inactivation. This, in turn, results in the accumulation of β-catenin in the cytosol, from where it can translocate to the nucleus and activate the expression of target genes (De Ferrari and Inestrosa 2000). A recent study also suggests that GSK-3β phosphorylation can be associated with phosphorylation of Akt at serine 473 (Ser473) residue, in the absence of Akt (Thr308) phosphorylation (Sutton and Rushlow 2011). Thus, there are a number of potential alternative regulators of GSK-3β which warrant investigation.

Here, we show that Akt (Ser473) phosphorylation is reduced in the D2R−/− CA3 neurons. This might explain the higher susceptibility to KA-induced damage observed in these neurons. Additionally, this vulnerability is p38MAPK- and Dvl-independent. Furthermore, β-catenin does not appear to be a downstream target of the GSK-3β pathway in D2R−/− CA3 neurons following KA, suggesting the involvement of alternative targets.

## Materials and Methods

### Animals and Seizure Induction

Experiments were performed in accordance with the European Communities Council Directive of 24 November 1986 (86/609/EEC). All possible measures were taken to avoid animals suffering at each stage of the experiments. All the mice used in this study were WT and D2R−/− littermates, obtained from heterozygote intermatings (F2 hybrids, C57BL/6 × 129/Sv mixed background) (Bozzi et al. 2000; Tripathi et al. 2010). Seizures were induced in male and female WT and D2R−/− adult mice (20–30 g, 3–5 months old) by intraperitoneal injection of KA (20 mg/kg). Previous studies from our laboratory showed no differences in susceptibility to seizures and seizure-induced excitotoxicity between genders in D2R−/− mice (Bozzi et al. 2000; Tripathi et al. 2010). Following injection, animals were returned to cages where seizure severity was assessed at 10-min intervals for up to 2 h, according to a modified Racine scale (Bozzi et al. 2000; Tripathi et al. 2010): stage 0, normal behaviour; stage 1, immobility; stage 2, forelimb and/or tail extension, rigid posture; stage 3, repetitive movements, head bobbing; stage 4, rearing and falling; stage 5, continuous rearing and falling; stage 6, severe whole-body convulsions; stage 7, death. Saline-injected animals of both genotypes were used as controls. Observers were blind to treatment and genotype of each animal and scores were assigned based on the maximum stage reached during the observational period. Twenty-four hours following KA injection, mice were sacrificed and brains processed for immunoblotting and immunohistochemistry as described below.

### Immunoblotting

Brains were immediately cooled for 5 s in liquid nitrogen. Whole hippocampi were dissected on an ice cold surface and flash frozen in liquid nitrogen. Proteins were extracted according to standard protocols established in the laboratory (Cotrufo et al. 2003) and total protein concentration measured with Nanodrop spectrophotometer (Thermo Scientific). Total protein extracts (20 μg; 30 μg for Dvl) were separated on 4–12 % NuPage Pre-Cast Gels (Invitrogen), transferred to nitrocellulose membranes and incubated with the following rabbit monoclonal antibodies: p38MAPK (1:500), phospho-p38MAPK (1:500), β-catenin (1:2,000), phosphorylated β-catenin (1:2,000)(all from Cell Signaling Technologies, MA, USA); Akt (1:500) and pAkt (Ser473) (1:500) (Abcam, UK); Dvl (1:10,000), β-tubulin (1:20,000) and GAPDH (1:8,000) (all from Santa Cruz, CA, USA). Membranes were then incubated with peroxidase-conjugated goat anti-rabbit secondary antibodies (1:10,000–20,000) (Santa Cruz, CA, USA). Protein levels were detected using chemiluminescence (BioRad) and quantitative densitometry using Image J software (http://rsb.info.nih.gov/ij/). β-Tubulin and GAPDH were used as a standard for protein quantification. Two to eight samples per group were analysed.

### Immunohistochemistry

Brains were fixed by immersion in 4 % PFA immediately following sacrifice. Vibratome sections (25 μm, coronal) were collected in serial order throughout the dorsal hippocampus and processed according to standard DAB immunohistochemistry protocol. Briefly, following quenching of endogenous peroxidases and blocking in 5 % goat serum in 0.1 % PB-T, slices were incubated in primary antibody for GSK-3β (1:100) pGSK-3β (1:500), Akt (1:500) pAkt (Ser473)(1:500), p38MAPK (1:80), phospho-p38MAPK (1:100), β-catenin (1:100) and Dvl (1:50). Bound antibody was detected by incubation with biotinylated goat anti-rabbit secondary antibody (1:1,000, Vector Lab, CA, USA) followed by incubation with DAB. Images were acquired by using a bright-field Nikon microscope at ×10 and ×20 primary magnifications. All immunohistochemical analyses were performed on two to five mice per group (one to three sections per animal per treatment group) with quantitative densitometry performed using Image J software. Briefly, images were converted to greyscale and inverted, mean grey value was calculated for the CA3 subregion. Results were expressed relative to saline-treated WT animals. Negative controls (slices incubated only with biotinylated goat anti-rabbit secondary antibody) did not reveal any detectable signal (data not shown).

### Statistical Analysis

All data are expressed as mean ± SEM. Statistical analysis was performed using the SigmaStat software. Analysis of seizure scores was performed by applying a two-way repeated measures ANOVA followed by post hoc Holm–Sidak test. Analysis of immunoblot and immunohistochemical data was performed by two-way ANOVA, followed by Bonferroni’s and Holm–Sidak multiple comparisons post hoc test, respectively.

## Results

### Increased Sensitivity of D2R−/− Mice to KA-Induced Seizures

**Fig. 1:**
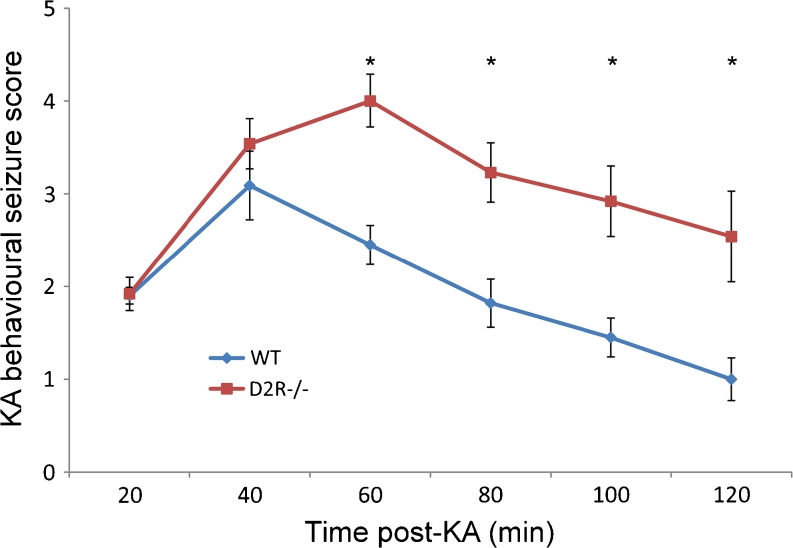
D2R−/− mice are more susceptible to KA-induced seizures. Analysis of mean behavioural seizure score at 20-min intervals following KA administration (20 mg/kg). D2−/− mice undergo more severe and sustained seizures (*n* = 11–13 per group). *p < 0.05 (WT vs D2R−/−, two-way RM ANOVA followed by Holm–Sidak multiple comparisons post hoc test)

WT and D2R−/− mice were monitored for behavioural seizures following KA injection. Focal seizures (stage 2) emerged in both WT (*n* = 11) and D2R−/− (*n* = 13) animals within 20 min of KA administration. At 40 min, WT animals experienced the most severe seizure score (3.1 ± 0.4) which subsequently subsided. D2R−/− animals, however, experienced more severe seizures (mean score 4.0 ± 0.3 at 60 min) and maintained significantly higher seizure activity for the duration of the 120-min observational period (Fig. 1). No mortality was observed in either group in the 24 h following KA injection. These results were in agreement with our previous data (Bozzi et al. 2000; Tripathi et al. 2010).

### Effects of KA-Induced Seizures on the p38MAPK Pathway

**Fig. 2:**
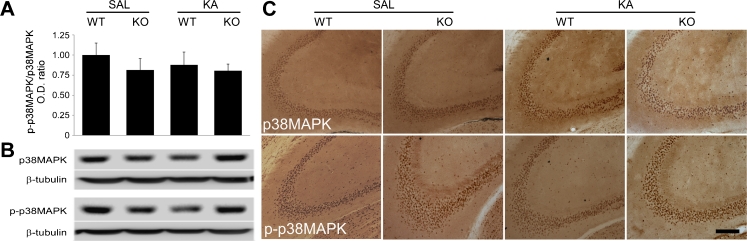
Hippocampal expression of p38MAPK and phosphorylated-p38MAPK following KA seizures in WT and D2R−/− mice. **a** Immunoblot analysis of phosphorylated p38 MAPK (p-p38MAPK) versus p38MAPK in the hippocampus from WT and DR2−/− (KO) mice, 24 h following saline (SAL) and KA treatment (*n* = 3 per group). There was no significant difference in expression levels between any of the groups. **b** Representative immunoblots from KA and saline-treated mice (*n* = 1 per lane). GAPDH is included as a loading control. **c** Representative photomicrographs (×10 magnification) showing the CA3 subregion labelled for p38MAPK and p-p38MAPK. *Scale bar* 250 μm

Previous data from our group showed a marked downregulation of GSK-3β phosphorylation in the hippocampus of D2R−/− mice following systemic KA injection (Tripathi et al. 2010). Here, we first investigated the hippocampal expression of p38MAPK and phosphorylated p38MAPK in WT and D2R−/− mice after systemic KA injection. Phosphorylation by p38MAPK has been proposed as an alternative mechanism for the inactivation of GSK-3β (Thornton et al. 2008). Immunoblotting experiments revealed no altered pattern of p38MAPK and phosphorylated p38MAPK (p-p38MAPK) levels in WT or D2R−/− saline-treated animals (*n* = 3 per group) (Fig. 2). Similarly, the levels of p38MAPK and p-p38MAPK were also unaltered 24 h following KA administration in WT or D2R−/− mice. Quantification of immunoblotting experiments, as revealed by separate measurement of p38MAPK and p-p38MAPK bands (data not shown) or by the ratio of p-p38MAPK/p38MAPK, showed no difference between the four experimental groups (Fig. 2). Immunohistochemistry experiments (performed on *n* = 2–6 mice per group) confirmed these findings, suggesting that p38MAPK signalling in the hippocampus is not affected by the induction of seizures.

### Effects of KA-Induced Seizures on Wnt Pathway Signalling

**Fig. 3:**
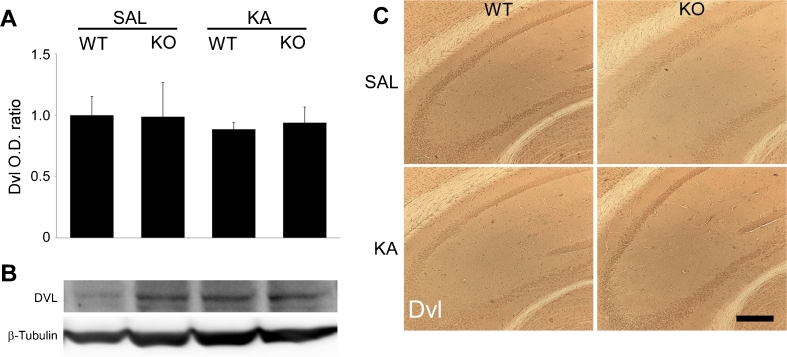
Hippocampal expression of Dvl following KA seizures in WT and D2R−/− mice. **a** Quantification of immunoblots for Dvl expression in hippocampi from WT and D2R−/− animals 24 h post-KA-induced seizures (*n* = 5–7 per group). No significant difference in expression was observed between the groups. **b** Representative immunoblots of hippocampal Dvl expression in WT and D2R−/− animals following saline and KA treatment (*n* = 1 per lane). β-Tubulin is included as a loading control. **c** DAB immunohistochemistry images (×10 magnification) of Dvl expression in the CA3 hippocampal subregion 24 h post-KA-induced seizures in WT and D2R−/− mice. *Scale bar* 250 μm. Abbreviations as in Fig. 2

**Fig. 4:**
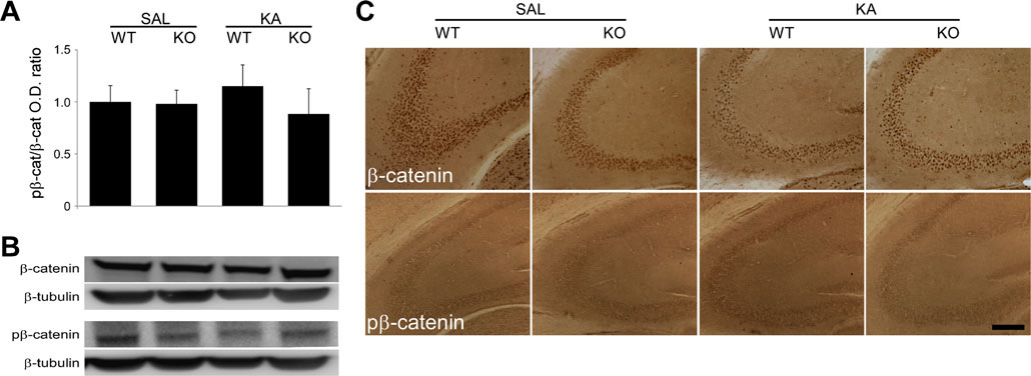
Hippocampal expression of β-catenin following KA seizures in WT and D2R−/− mice. **a** Quantitative western blot analysis of phosphorylated β-catenin (pβ-catenin) versus total β-catenin in the hippocampus of WT and D2R−/− mice, 24 h post-KA-induced seizures (*n* = 4 per group). No significant difference in expression was observed between the groups. **b** Representative immunoblots of pβ-catenin and β-catenin expression in WT and D2R−/− hippocampi following saline and KA treatment (*n* = 1 per lane). β-Tubulin is included as a loading control. **c** DAB immunohistochemistry images (×10 magnification) of β-catenin and pβ-catenin expression in the CA3 hippocampal subregion 24 h post-KA-induced seizures in WT and D2R−/− mice. *Scale bar* 250 μm. Abbreviations as in Fig. 2

GSK-3β has been described to have an important role in the Wnt pathway, being inactivated following activation of Dvl (Inestrosa and Arenas 2010). To assess the role of Wnt signalling in the increased susceptibility to seizure-induced neurodegeneration in the D2R−/− hippocampus, we analysed whether Dvl levels were altered following KA treatment of WT and D2R−/− mice. Neither immunoblotting (*n* = 5–7 per group) nor immunohistochemistry (*n* = 2–6 per group) revealed any difference between WT and D2R−/− animals 24 h following saline or KA treatment (Fig. 3). β-Catenin expression, known to be activated downstream of GSK-3β in the Wnt pathway, was not altered in our model. Indeed, immunohistochemistry revealed no alteration in β-catenin or phosphorylated β-catenin levels in the CA3 of either WT or D2R−/− animals 24 h following saline or KA treatment (*n* = 2–6 per group). These results were confirmed by immunoblotting analyses for β-catenin and phosphorylated β-catenin (*n* = 4 per group) (Fig. 4). Taken together, these data suggest that the altered GSK-3β activation following KA-induced seizures is independent of the Wnt pathway.

### Downregulation of pAkt (Ser473) in the CA3 Following KA-Induced Seizures in D2R−/− Mice

**Fig. 5:**
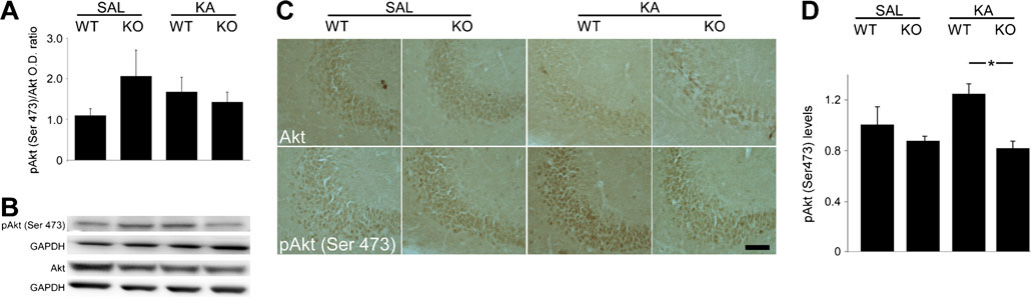
Hippocampal expression of Akt and pAkt (Ser473) following KA seizures in WT and D2R−/− mice. **a** Immunoblot quantification 24 h following KA-induced seizures reveal no significant difference in expression of pAkt (Ser473)/Akt following saline or KA treatment in WT or D2R−/− animals (*n* = 5–8 per group). **b** Representative immunoblots of hippocampal pAkt (Ser 473) and Akt expression in WT and D2R−/− animals following saline and KA treatment (*n* = 1 per lane). GAPDH is included as a loading control. **c** Representative photomicrographs (×20 magnification) of the CA3 subfield, showing upregulation of pAkt (Ser473) following KA-induced seizures in WT animals in the absence of a similar upregulation in D2R−/− animals. **d** Immunohistochemical quantification reveal upregulation of pAkt (Ser473) in the CA3 of WT animals which is significantly different to KO animals 24 h following KA-induced seizures (*n* = 2–5 per group). *Scale bar* 125 μm. Abbreviations as in Fig. 2. *p < 0.05 (WT vs D2R−/−, two-way ANOVA followed by Holm–Sidak multiple comparisons post hoc test)

**Fig. 6:**
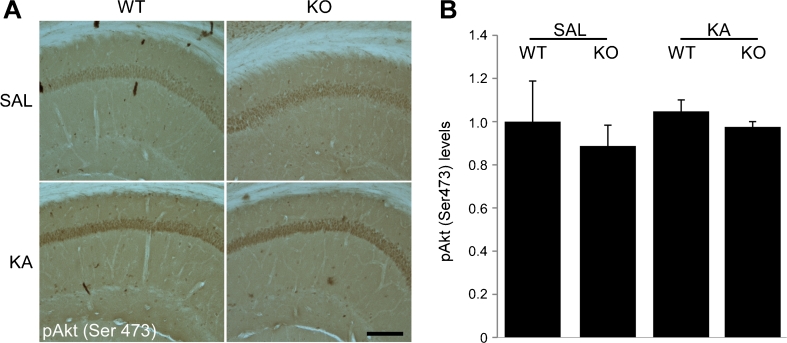
No downregulation of pAkt (Ser473) in the CA1 subfield following KA seizures in WT and D2R−/− mice. **a** Representative DAB immunohistochemistry images (×10 magnification) displaying maintenance of pAkt (Ser473) levels 24 h post-KA-induced seizures in the CA1 subregion in both WT and D2R−/− mice. **b** Immunohistochemical quantification 24 h following KA-induced seizures reveal no significant difference in expression of pAkt (Ser473) following saline or KA treatment in WT or D2R−/− animals (*n* = 2–5 per group). *Scale bar* 250 μm. Abbreviations as in Fig. 2

Our previous results had shown that hippocampal pAkt (Thr308) levels were not affected following KA-induced seizures (Tripathi et al. 2010), suggesting that a non-canonical pathway may be responsible for the activation of GSK-3β in the hippocampus of KA-treated D2R−/− mice. However, recent studies have proposed that D2R-mediated signalling might affect GSK-3β activity through phosphorylation of Akt on Ser473 (Sutton and Rushlow 2011). Immunoblots performed on protein lysates from whole hippocampi revealed no change in overall expression of pAkt (Ser473) 24 h following KA administration (*n* = 5–8 per group). However, immunohistochemical analyses performed at the same time point revealed an increase of pAkt (Ser473) in the CA3 region of WT mice following KA (*n* = 2–5 per group) (Fig. 5). Conversely, the vulnerable CA3 of D2R−/− animals displayed a downregulation of pAkt (Ser473). These results suggest that lack of D2R-mediated signalling affects Akt-dependent modifications not only on Thr308 as shown in the striatum (Beaulieu et al. 2011) but also on Ser473 in the hippocampus, which may, in turn, result in an aberrant regulation of GSK-3β in the CA3 region of D2R−/− mice. Furthermore, the CA1 subregion, which is protected from neurodegeneration in both WT and D2R−/− mice (Bozzi and Borrelli 2006; Bozzi et al. 2000), maintained strong expression of pAkt (Ser473) following KA (Fig. 6) (*n* = 2–5 per group). The selective high expression levels of pAkt (Ser473) in the CA1 might also explain why we were unable to detect differences between WT and D2R−/− by western blot analyses. The observed changes in pAkt (Ser473) levels were independent of any changes in basal Akt expression in any of the experimental groups throughout the hippocampal subfields. Our results are in support of a potential role for Akt (Ser473) phosphorylation on neuronal survival, which is reduced in the vulnerable CA3 of the D2R−/− mice.

## Discussion

The present study shows that loss of D2R signalling in the CA3 region of D2R−/− mice leads to reduced (Ser473) phosphorylation of Akt (a crucial regulator of GSK-3β), thus rendering neurons more vulnerable to KA-induced damage. Conversely, other GSK-3β regulatory pathways, such as the p38MAPK and Wnt pathways, do not appear to be involved in this process.

Dopamine has been shown to play an important role in the regulation of seizure-induced cell death. While D1-like dopamine receptors have been shown to have a proconvulsant and proapoptotic effect, D2-like dopamine receptors play the opposite role, ameliorating seizure phenotype and reducing seizure-induced damage when activated (Bozzi and Borrelli 2006; Bozzi et al. 2011; Starr 1996). The canonical D2 receptor signalling pathway acts via G_i/o_-coupled proteins to decrease adenylate cyclase and, in turn, reduce cAMP production (Callier et al. 2003). However, in the last decade, the regulation of the Akt/GSK-3β pathway by dopamine has become an area of increasing interest. Activation of D2R causes the dephosphorylation (inactivation) of pAkt (Thr308), followed by dephosphorylation (activation) of GSK-3β (Beaulieu et al. 2005, 2007a). Activity of Akt and GSK-3β was unaltered following direct modulation of cAMP levels indicating that this signalling is cAMP-independent (Beaulieu et al. 2004). Once activated, GSK-3β has been shown to play a role in neurodegeneration (Gomez-Sintes et al. 2011). We previously observed an increase of GSK-3β activity (dephosphorylation) in the D2R−/− hippocampus 24 h after KA-induced seizures (Tripathi et al. 2010). This change in activity was not accompanied by a change in pAkt (Thr308) phosphorylation, suggesting that an alternative pathway may be active in the hippocampus.

The best characterised mechanism of inactivation of GSK-3β is by Akt, through phosphorylation of the N-terminus at Ser9 (Cross et al. 1995). However, phosphorylation of Akt within the C-terminus at Ser473 has recently been implicated in the regulation of GSK-3β in D2R signalling cascade (Sutton and Rushlow 2011). In the present study, we observed that KA seizures produced a robust upregulation of pAkt (Ser473) in the WT CA3 but not in the vulnerable CA3 of D2R−/− mice. In addition, in the undamaged CA1 subregion of both genotypes, there was maintenance of high levels of pAkt (Ser473). These data are in agreement with other studies reporting the expression levels of pAkt (Ser473) following brain injury. A downregulation of pAkt (Ser473) was reported at the impact site in a model of traumatic brain injury while adjacent areas which remained undamaged displayed an upregulation of pAkt (Ser473) (Noshita et al. 2002). Furthermore, models of global and focal ischemia demonstrate a decrease in pAkt (Ser473) in the ischemic core along with an increase in pAkt (Ser473) in undamaged cells (Noshita et al. 2001; Yano et al. 2001). Transient upregulation of pAkt in the CA1 following global ischemia has also been described in delayed ischemia-induced cell death (Kawano et al. 2001) and inhibition of pAkt during ischemic preconditioning eliminates the neuroprotection afforded by the preconditioning stimulus (Yano et al. 2001). This suggests that the observed response may be dependent on the severity of the stress stimulus. These data support a role for pAkt (Ser473) in cell survival following brain insult. In the current model, it appears that the absence of the D2R pathway in the CA3 of D2R−/− mice prevents pAkt (Ser473) upregulation in response to KA challenge; this could be consistent with the enhanced vulnerability of CA3 pyramidal neurons to excitotoxic damage. Since, at low KA doses (20 mg/kg), WT mice with a C57Bl/6-derived genetic background are resistant to seizure-induced damage (Bozzi et al. 2000; Schauwecker and Steward 1997), the absence or reduction of pAkt (Ser473) signalling may promote the more vulnerable state observed in D2R−/− mice. Conversely, increased levels of level of pAKT in D2R−/− mice might have a protective effect. Increased levels of pAkt have been detected in the striatum (Beaulieu et al. 2007b) but not in the hippocampus (this study, Fig. 5; Tripathi et al. 2010) under basal conditions. Thus, it would be interesting to investigate the neuroprotective effect of pharmacological upregulation of pAkt, obtained for example by neurotrophin receptor activation (Iwakura et al. 2008)

Contrary to what is described in this study, dephosphorylation of pAkt (Ser473) has been reported 24 h following KA (30 mg/kg) in whole hippocampal protein extracts (Crespo-Biel et al. 2007). This may be explained by the different pattern of damage in the two models. Crespo-Biel et al. describe a diffuse hippocampal damage including the CA3, DG and CA1 [Crespo-Biel et al. 2007], while in the current model only a small subset of CA3 neurons are vulnerable to the insult (Bozzi and Borrelli 2006). Some evidence suggests phosphorylation of Akt can occur more rapidly after KA treatment: an acute upregulation of pAkt (Ser473) has been observed 15 min following systemic KA (30 mg/kg) administration in vivo, which returns to control levels after 4 h. This was associated with a concomitant upregulation of pGSK-3β (Thr308) at 15 min, which also returned to control levels after 4 h (Rojo et al. 2008). Accordingly, it may be of interest in the current model to examine earlier time points, where an even more robust alteration in pAkt (Ser473) activity might be observed.

We exclude that the downregulation of pAkt (Ser473) detected in the CA3 subfield of D2R−/− mice is due to a massive cell death in this area. First, we previously showed that the KA dose used in this study (20 mg/kg) induces cell death only in a very small subset of CA3 cells in D2R−/− mice, and this degeneration is detectable only after 5 days from KA administration (Bozzi et al. 2000). Indeed, cresyl violet staining of hippocampal sections from D2R−/− 24 h following KA administration revealed a very limited cell damage in the CA3 area (data not shown). Second, our previous results showed that hippocampal pAkt (Thr308) levels were not affected following KA-induced seizures (Tripathi et al. 2010), suggesting that the downregulation of pAkt (Ser473) is specific and not related to cell loss.

p38MAPK is part of a signalling cascade that has been implicated in the neuronal death associated with KA-induced seizures (Herlaar and Brown 1999; Park et al. 2008), with expression peaking between 1 and 4 days post-seizures (Kim et al. 2004). p38MAPK has also been implicated as an alternative regulator of GSK-3β phosphorylation (Thornton et al. 2008). The absence of any alteration in expression and phosphorylation of p38MAPK suggests that the GSK-3β activation observed in KA-treated D2R−/− mice (Tripathi et al. 2010) is p38MAPK-independent. Previous studies have shown phosphorylation of p38MAPK peaks 1 day following KA (Kim et al. 2004), which is the same time analysed in the current experiments. Again, this may result from the milder insult observed in the present study (CA3 damage only in D2R−/− mice), while the induction of p38MAPK activity is associated with severe damage in the CA3 and mild damage in the CA1 (Kim et al. 2004).

Finally, we investigated two key members of the Wnt signalling pathway to reflect potential activation of GSK-3β and cell death seen in KA-treated D2R−/− mice. The Wnt pathway has been identified as one of the key regulators of GSK-3β activity in a number of pathological conditions (Inestrosa and Arenas 2010) and may play a role in KA seizure-induced damage. Following activation of the cell surface receptor Frizzled, Dvl is activated and leads to the activation of a complex including Axin, APC and GSK-3. Under basal conditions, this complex acts to phosphorylate β-catenin, which in turn leads to its degradation. However, when activated, the complex prevents β-catenin degradation allowing it to accumulate in the cytosol, translocate to the nucleus and promote the expression of target genes. In the current study however, no change in total Dvl expression was observed. Recent studies suggest that D2R could regulate Akt activity specifically by Dvl-3 and independent of Dvl-1 and Dvl-2 (Sutton and Rushlow 2011). Further investigation into the specific activities of the different Dvl homologs may provide clarification on any involvement of Dvl.

Increased total and dephosphorylated (active) β-catenin have been shown to result in death of dopaminergic neurons (Rawal et al. 2009), with alterations in β-catenin phosphorylation being linked to neurodegeneration in models of Alzheimer’s disease (Lucas et al. 2001). The role of β-catenin in seizure-induced cell death remains poorly investigated. One study reported the induction of β-catenin in the hippocampus of rats which underwent massive brain damage after systemic KA, while rats with no damage showed no change in β-catenin expression (Busceti et al. 2007). Once again, the absence of β-catenin regulation in the KA-treated D2R−/− mice may be a result of the CA3-restricted hippocampal damage experienced by these animals (Bozzi et al. 2000).

Further experiments are required to fully elucidate the role of Akt /GSK-3β signalling to CA3 neuronal death in the current model. Recent advances in animal models have proved an important tool in investigating GSK-3β deregulation in neuronal death (for review, see Gomez-Sintes et al. 2011). Employment of mouse models involving downregulation, upregulation or modulation of GSK-3b could demonstrate a functional role for GSK-3β in the neuronal death observed in the current model.

We propose that the neurodegeneration observed in the CA3 of D2R−/− mice following a low dose of KA may result from the reduced neuroprotective response normally afforded by upregulation of pAkt (Ser473), and this effect is independent of p38MAPK and Dvl activity. In addition, β-catenin expression is not altered in the hippocampus of KA-treated D2R−/− mice, suggesting further investigation is required to fully elucidate the targets of GSK-3β (such as Tau; De Ferrari and Inestrosa 2000; Hartigan and Johnson 1999) downstream of D2R that may be involved in dopamine-dependent response to excitotoxicity.
